# Physical and chemical changes of rapeseed meal proteins during toasting and their effects on *in vitro* digestibility

**DOI:** 10.1186/s40104-016-0120-x

**Published:** 2016-10-18

**Authors:** Sergio Salazar-Villanea, Erik M. A. M. Bruininx, Harry Gruppen, Wouter H. Hendriks, Patrick Carré, Alain Quinsac, Antonius F. B. van der Poel

**Affiliations:** 1 Wageningen Livestock Research, P.O. Box 338, 6700 AH Wageningen, The Netherlands; 2 Animal Nutrition Group, Wageningen University & Research, P.O. Box 338, 6700 AH Wageningen, The Netherlands; 3 Agrifirm Innovation Center, Royal Dutch Agrifirm Group, P.O. Box 20018, 7302 HA Apeldoorn, The Netherlands; 4 Laboratory of Food Chemistry, Wageningen University & Research, P.O. Box 17, 6700 AA Wageningen, The Netherlands; 5 CREOL/OLEAD, 11 rue Monge, Parc Industriel, 33600 Pessac, France; 6 Terres Inovia, 11 rue Monge, Parc Industriel, 33600 Pessac, France

**Keywords:** Hydrolysis rate, *In vitro* protein digestibility, Rapeseed meal, Reactive lysine, Secondary structure

## Abstract

**Background:**

Toasting during the production of rapeseed meal (RSM) decreases ileal crude protein (CP) and amino acid (AA) digestibility. The mechanisms that determine the decrease in digestibility have not been fully elucidated. A high protein quality, low-denatured, RSM was produced and toasted up to 120 min, with samples taken every 20 min. The aim of this study was to characterize secondary structure and chemical changes of proteins and glucosinolates occurring during toasting of RSM and the effects on its *in vitro* CP digestibility.

**Results:**

The decrease in protein solubility and the increase of intermolecular β-sheets with increasing toasting time were indications of protein aggregation. The contents of NDF and ADIN increased with increasing toasting time. Contents of arginine, lysine and *O*-methylisourea reactive lysine (OMIU-RL) linearly decreased with increasing toasting time, with a larger decrease of OMIU-RL than lysine. First-order reactions calculated from the measured parameters show that glucosinolates were degraded faster than lysine, OMIU-RL and arginine and that physical changes to proteins seem to occur before chemical changes during toasting. Despite the drastic physical and chemical changes noticed on the proteins, the coefficient of *in vitro* CP digestibility ranged from 0.776 to 0.750 and there were no effects on the extent of protein hydrolysis after 120 min. In contrast, the rate of protein hydrolysis linearly decreased with increasing toasting time, which was largely correlated to the decrease in protein solubility, lysine and OMIU-RL observed. Rate of protein hydrolysis was more than 2-fold higher for the untoasted RSM compared to the 120 min toasted material.

**Conclusions:**

Increasing the toasting time for the production of RSM causes physical and chemical changes to the proteins that decrease the rate of protein hydrolysis. The observed decrease in the rate of protein hydrolysis could impact protein digestion and utilization.

## Background

Rapeseed meal (RSM) is the most important protein source utilized in commercial swine and poultry diets after soybean meal [1–3]. The production process of RSM involves toasting to remove the organic solvent remaining after solvent extraction of the oil and to inactivate antinutritional factors present such as glucosinolates [4]. Direct application of live steam is used during toasting to complete the solvent removal, which also increases the moisture content. The toasting process time usually ranges from 60 to 90 min at 100–110 °C, which can increase the variation in the lysine content and ileal digestibility of most AA in RSM [4, 5]. The coefficient of variation of the apparent ileal digestibility of lysine in poultry increased from 1.4 % in the solvent extracted meal to 5.4 % after toasting [5].

Both physical and chemical changes of proteins due to thermal processing can influence protein digestibility [6]. Autoclaving at 120 °C for 20 min increased the proportion of random coil in the secondary protein structure of legume seeds, which is related to protein denaturation and increased CP *in vitro* digestibility [7]. At the same time, appearance of intermolecular β-sheets, linked to decreased protein digestibility, was reported in the same study. The net effect on the *in vitro* crude protein (CP) digestibility seems to be related to the ratio between both types of physical changes. Chemical changes can be the result of Maillard reactions or covalent crosslinking between amino acids (AA). Increasing the toasting time decreased the lysine and reactive lysine contents [8]. In addition, the standardized ileal digestibility of CP was reduced from 66 to 60 % and that of lysine from 64 to 54 % when the toasting time was increased from 48 to 93 min [8]. Chemical changes due to Maillard reactions were suggested to be responsible for the decrease in protein and amino acid digestibility. However, chemical changes of proteins due to Maillard reactions do not completely explain the reduction in the digestibility of all AA, as observed in that study. This suggests that also changes in the structure of proteins (e.g. secondary and tertiary) affect the digestion process.

The aim of the present experiment was to characterize the physical and chemical changes that occur to rapeseed proteins during toasting and the influence of these changes on *in vitro* CP digestibility. We hypothesize that increasing toasting times causes physical and chemical changes to rapeseed proteins, resulting in decreased *in vitro* CP digestibility.

## Methods

### Materials

A batch of commercially available winter 00-rapeseed (*Brassica napus*), harvested in the southwest of France in 2013, was used. All chemicals used were of analytical grade. Pepsin (2,000 FIP U/g) was obtained from Merck (Darmstadt, Germany), whilst pancreatin (grade IV from porcine pancreas), trypsin (type IX-S, 13,000–20,000 BAEE units/mg protein), chymotrypsin (type II, >40 units/mg protein), and peptidase from porcine intestinal mucosa (50–100 units/g solid) were obtained from Sigma-Aldrich (St Louis, MO, USA).

### Rapeseed meals preparation

**Fig. 1:**
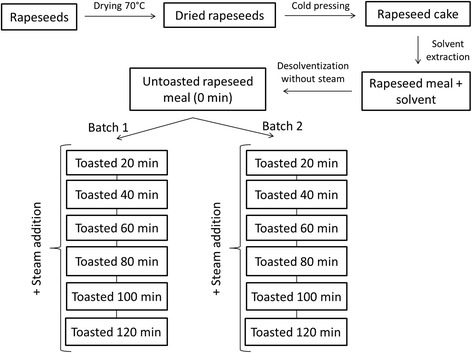
Schematic view of the experimental rapeseed treatment

All processing of the rapeseed was conducted at the pilot plant of CREOL (Pessac, France). The batch of rapeseed was dried in a warm-air dryer at 70 °C to a moisture level of 5 % (w/w) (Fig. 1). After drying, the rapeseed was cold pressed (La Mecanique Moderne MBU 75 type, Arras, France) at 250 kg/h. Temperatures during pressing did not exceed 80 °C. Continuous extraction of the cake by hexane was performed on a belt extractor (B-1930, Desmet-Ballestra, Zaventem, Belgium) at 160 kg/h flow of the cake and 230 L/h flow of solvent. Temperature of the rapeseed cake during solvent extraction did not exceed 55 °C. The solvent remaining after solvent extraction of the oil was removed using indirect heat (i.e. without use of direct live steam) in a desolventizer-toaster (Schumacher type, Desmet-Ballestra, Zaventem, Belgium; 6 trays with a rotating arm and 1 m internal diameter) for 60 min. Temperatures during desolventization were 90 ± 3 °C. Spot samples (5 kg) were obtained after drying, cold-pressing, hexane extraction, and desolventization with indirect heat.

A batch of 150 kg of the desolventized-untoasted RSM was toasted in the lower tray of the desolventizer-toaster with injection of live steam (30 kg/h), whilst indirect steam pressure was set at 3 bars and arm rotations at 20 rpm. These conditions were maintained for 120 min, with spot samples (5 kg) taken every 20 min through a door in the desolventizer-toaster. Monitoring of time was initiated when the temperature inside the desolventizer-toaster reached 100 °C. A second batch of 150 kg of the desolventized-untoasted RSM was used for duplication of the toasting experiment on the next day (Fig. 1). The samples obtained from both toasted batches (in total 12 toasted RSM plus the untoasted RSM) were analyzed separately. Temperatures during toasting ranged from 107 to 112 °C on the first day and from 109 to 112 °C on the second day.

### Analytical methods

The desolventized RSM and the toasted RSMs were ground to pass a 1 mm sieve using a centrifugal mill (ZM200, Retsch, Haan, Germany) at 8,000 rpm prior to chemical analysis. Samples (5 g) were re-ground using a ball (Ø 12 mm) mill (MM2000, Retsch) at a frequency of 80 during 3 min and used for secondary structure, degree of denaturation, amino acid and reactive lysine analysis. All chemical and secondary structure analyses were performed in duplicate, except for reactive lysine, which was performed in triplicate.

#### Nutrient contents

Dry matter (DM) content was determined by oven-drying at 103 °C to constant weight according to ISO 6496 [9]. Nitrogen content was analyzed by combustion according to AOAC 968.06 (Thermo Quest NA 2100 Nitrogen and Protein Analyzer; Breda, the Netherlands) [10]. Nitrogen content in the nitrogen solubility index (NSI) and nitrogen linked to the acid detergent fiber (ADIN) determinations were measured using the Kjeldahl method according to ISO 5983 [11]. A conversion factor of 6.25 was used for the calculation of CP content from nitrogen. Crude fat content was determined according to ISO 734–2 [12]. The neutral detergent fiber (NDF) and acid detergent fiber (ADF) contents were determined using a fiber analyzer equipment (Fiber Analyzer, Ankom Technology, Macedon, NY) according to a modification of the procedure of Van Soest et al. [13]. The NDF determination involved enzymatic incubation with α-amylase and alcalase, but without addition of sodium sulfite. The ADIN content was determined in the residues after hydrolysis with acid detergent reagents.

For the determination of amino acid content, samples were hydrolyzed with 6 mol/L HCl at 110 °C for 23 h and the hydrolysates were adjusted to pH 2.2 using NaOH. Amino acids were determined by post column reaction with ninhydrin, after separation by ion exchange chromatography. Photometric detection was performed at 570 nm and at 440 nm for proline according to ISO 13903 [14]. Norleucine was used as an internal standard.

Reactive lysine was determined using a method described by Moughan and Rutherfurd [15]. In short, reactive lysine was converted into homoarginine by incubation with *O*-methylisourea (OMIU) for 7 d. Samples were subsequently hydrolyzed with 6 mol/L HCl at 110 °C for 23 h and the hydrolysates were adjusted using NaOH to pH 2.2. After separation by ion exchange chromatography, the homoarginine content was determined by post column reaction with ninhydrin using photometric detection at 570 nm. The amount of OMIU-reactive lysine (OMIU-RL) was calculated from the molar amount of homoarginine and the molecular weight of lysine.

Glucosinolates were quantified according to ISO 9167–1 [16]. Glucosinolates were extracted by a 70 % (v/v) methanol in water mixture at 70 °C, using sinigrin as internal standard. The glucosinolates were then linked on an anion-exchange column, purified and on-column desulphated by overnight action of sulphatase enzyme. Desulphoderivatives were eluted with water and analyzed using reverse phase liquid chromatography with gradient elution and UV detection at 229 nm.

#### Differential scanning calorimetry

Degree of denaturation of the samples was studied using differential scanning calorimetry (DSC12E, Mettler-Toledo, Greifensee, Switzerland). Samples (15–20 mg) were weighed into medium pressure crucibles (ME-29990, Mettler-Toledo, Greifensee, Switzerland) and approximately 60 mg of demineralized water was added. Samples were left overnight to equilibrate at 4 °C. The heating program ranged from 15 to 120 °C at a rate of 5 °C/min, with an initial isothermal step of 5 min at 15 °C. A crucible filled with demineralized water was used in the reference cell. Enthalpy (J/g CP) of denaturation was determined using the TA89E software (Version 3, Mettler-Toledo, Switzerland) for analysis of thermo-analytical data.

#### Protein solubility

Protein dispersibility index (PDI) in water was measured using a modification of the method of AOCS [17]. Approximately 75 mg of sample was weighed and mixed for 30 s with 1.5 mL of water in a vortex. The sample was then mixed for 20 min in a rotator SB2 (Stuart-Barloworld Scientific Staffordshire, UK) with an angle of 90° and a speed of 20 rpm. Centrifugation was performed at 13,000 × g for 10 min at room temperature and the supernatant analyzed for nitrogen content. Soluble protein in 0.2 % (w/v) KOH, equivalent to nitrogen solubility index (NSI), was measured according to ISO 14244 [18].

#### Protein secondary structure

Attenuated total reflectance Fourier transform infrared spectroscopy (ATR-FTIR) (Tensor 27, Bruker, MA, USA) was used to measure the spectra ranging from 600 to 4,000/cm. The spectra were measured as absorbance with a resolution of 4/cm and using 16 scans per spectra. Spectral measurements were performed in duplicate and corrected for background. The OPUS software Version 7.2 (Bruker, Billerica, MA, USA) was used for all spectral transformations and calculations according to the procedure described by Hu et al. [19] with minor modifications. Briefly, Fourier self-deconvolution was applied to the Amide I region (1,595–1,705/cm) of the original spectra, using a Lorentzian correction with a bandwidth of 25/cm and a noise reduction factor of 0.3. The second derivative was applied to the original spectra and used for peak selection. Curve fitting of the selected peaks was performed using a Gaussian approximation with the Levenberg-Marquardt algorithm as the fitting method and an iteration time of 10 s. Selected peaks were identified using existing literature [7, 20].

### *In vitro* digestibility

#### Two-step enzymatic digestibility

Dry matter and CP digestibility were determined using a modification of the method from Boisen and Fernández [21]. Briefly, 5 g of material was mixed with 125 mL of a pH 6.0 disodium phosphate buffer (0.1 mol/L) and 50 mL of 0.2 mol/L HCl. This mixture was incubated with 5 mL of a freshly prepared pepsin solution (0.025 g/mL) for 2 h at 39 °C and pH 2.0. After this incubation, 50 mL of pH 6.8 sodium phosphate buffer (0.2 mol/L) and 25 mL of 0.6 mol/L NaOH were added. The pH was adjusted to 6.8, 5 mL of a freshly prepared pancreatin solution (0.10 g/mL) were added and the mixture incubated for 4 h. All buffers and solutions were preheated at 39 °C before addition, with the exception of the enzyme solutions. After the latter incubation, 5 mL of a 20 % (w/v) sulfosalicylic acid solution was added and the mixture centrifuged at 4,500 × g for 10 min at room temperature. The insoluble residue was collected, freeze-dried and analyzed for dry matter and nitrogen content.

#### pH-STAT enzymatic hydrolysis

Enzymatic hydrolysis was performed with the addition of porcine trypsin, bovine chymotrypsin and porcine intestinal peptidase using a modification of the pH-STAT method from Pedersen and Eggum [22]. In contrast to the original method, the hydrolysis was extended for 120 min after the addition of the enzymes. The volume of 0.1 mol/L NaOH added was used for the calculation of the degree of hydrolysis (DH) according to Eq. 1:

1 $$ \mathrm{D}\mathrm{H} = \left(Vb\times Nb\right)/\left(\upalpha \times mp\times \mathrm{htot}\right) $$

in which *Vb* is the volume of NaOH solution added, *Nb* is the normality of the titration solution, α is the degree of dissociation of the α-NH_2_ group (i.e. 0.794 at 37 °C and pH 8), *mp* is the mass of protein in grams and *htot* the total number of peptide bonds per gram of substrate (7.8 eq/g). The DH was used to calculate the rate of protein hydrolysis (*k*) based on the model described by Butré et al. [23] shown in Eq. 2:

2 $$ \mathrm{D}\mathrm{H} = 1/b\times ln\left(k\times ht+1\right) $$

In this model *b* is a parameter that defines the shape of the curve, *k* is the constant for the rate of protein hydrolysis (/s) and *ht* is the hydrolysis time (s). The model was fitted using the MODEL procedure of SAS software (Version 9.3, SAS Institute Inc., Cary, NC).

### Calculations and statistical analysis

Degradation rate constants and half-life for the parameters were calculated according to first-order reactions which were selected after fitting zero and second order reactions. Regression equations for the effect of toasting time were generated using the GLM procedure of the statistical software SAS. Correlations between parameters related to protein changes (e.g. NSI, PDI, lysine and OMIU-RL content, secondary structure) and *in vitro* digestibility (e.g. CP digestibility, DH after 120 min, *k*) were determined using the CORR procedure of SAS software. Linear or quadratic effects were considered as significant when the *P*-values were lower than 0.05 and as trends when *P*-values were between 0.05 and 0.10. The experimental unit was the RSM at each toasting time point.

## Results

During the oil extraction process of the rapeseed seeds, the crude fat content was reduced from 493 g/kg DM in the seeds to 16 g/kg DM in the untoasted meal (Table 1). At the same time, the NSI was decreased from 86.9 % in the seed to 79.9 % in the untoasted meal.

**Table 1:** Characterization of rapeseed samples before and after toasting^a^

Material	DM, g/kg	CP, g/kg DM	Crude fat, g/kg DM	NDF, g/kg DM	ADF, g/kg DM	ADIN, g/kg DM	Denaturation enthalpy, J/g CP	NSI, g/kg CP	PDI, g/kg CP
Rapeseed	937	-	493	-	-	-	2.23	869	-
Dried rapeseed	947	-	489	-	-	-	-	861	-
Rapeseed cake	923	-	135	-	-	-	-	861	-
RSM + solvent	907	-	13	-	-	-	-	825	-
Toasting time RSM									
0 min	913.0	360.0	16	274.4	217.4	3.0	2.34	799.0	260.7
20 min	917.7	362.0	-	278.2	211.7	3.0	1.29	695.5	161.8
40 min	918.9	366.3	-	291.4	215.5	3.1	1.23	597.5	129.3
60 min	922.8	372.9	-	319.1	213.3	3.1	1.13	537.0	105.3
80 min	924.6	368.4	-	338.9	216.0	3.3	1.07	513.0	84.7
100 min	917.8	363.7	-	354.6	217.1	3.4	1.03	475.5	72.6
120 min	930.8	369.1	-	365.3	218.2	3.8	0.74	431.0	63.3
SEM	1.7	1.4		9.8	1.1	0.3	0.11	31	15
*P*-value									
Linear	0.02	0.19	-	<0.001	0.21	<0.001	0.02	<0.001	<0.001
Quadratic	0.90	0.15	-	0.91	0.40	0.02	0.07	<0.001	<0.001

^a^ *DM* dry matter, *CP* crude protein, *NDF* neutral detergent fiber, *ADF* acid detergent fiber, *ADIN* nitrogen linked to the acid detergent fiber, *NSI* nitrogen solubility index, *PDI* protein dispersibility index, *RSM* rapeseed meal, *SEM* standard error of the mean

There was no effect of toasting time on the CP content of RSM, whilst there was a linear increase (*P* = 0.02) of the DM content with increasing toasting time (Table 1). There was a 33 % linear increase (*P* < 0.001) in the NDF content with increasing toasting time from the untoasted to the 120 min toasted RSM. In contrast, the ADF content was not affected (*P* > 0.05) by toasting time. Linear (*P* < 0.001) and quadratic (*P* = 0.02) effects of toasting time were found on the content of ADIN. The increase in the content of ADIN was more evident after 60 min toasting. Toasting time had a linear effect (*P* = 0.02) and a tendency for a quadratic effect (*P* = 0.07) on the denaturation enthalpy, which decreased with increasing toasting time. There were linear (*P* < 0.001) and quadratic (*P* < 0.001) effects of toasting time on NSI and PDI, with more apparent effects at low toasting times.

### Glucosinolates content

There were linear and quadratic effects of toasting time on the content of total (*P* = 0.001), alkenyl (*P* < 0.01) and indolyl plus aralkyl (*P* < 0.001) glucosinolates (Table 2). The largest decrease seems to occur after 60–80 min of toasting. Not all the glucosinolate types, however, responded to toasting in the same manner. Whilst the contents of epi-progoitrin, sinalbin and neoglucobrassicin were linearly reduced (*P* < 0.001) with increased toasting times, the effect of toasting time was both linear and quadratic (*P* < 0.05) for the other glucosinolates. The most abundant alkenyl glucosinolates was progoitrin (Table 2) which, even after toasting for 120 min, remained present at 9 % of its content in the untoasted RSM. Gluconapoleiferin was the most resilient alkenyl glucosinolate after toasting, as 16 % of the content of the untoasted RSM can still be found after 120 min toasting.

**Table 2:** Content (μmol/g DM) of glucosinolates in rapeseed meal samples toasted for different times^a^

Toasting time	PRO	EPRO	GNL	GNA	GBN	SNB	GST	4-OHGBS	GBS	NGBS	Alk	Ara + Ind	Total
0 min	12.62	0.36	0.81	4.83	2.66	0.23	0.63	5.50	0.23	0.11	21.28	6.70	27.98
20 min	10.18	0.28	0.68	4.01	2.08	0.25	nd	2.69	0.18	0.09	17.21	3.20	20.41
40 min	8.15	0.25	0.56	3.22	1.67	0.18	nd	1.39	0.13	0.08	13.83	1.77	15.60
60 min	5.94	0.16	0.41	2.47	1.11	0.16	nd	0.58	0.09	0.06	10.09	0.89	10.97
80 min	3.84	0.06	0.28	1.65	0.71	nd	nd	0.20	0.06	nd	6.52	0.26	6.78
100 min	2.27	nd	0.19	0.99	0.36	0.03	nd	0.06	nd	nd	3.81	0.08	3.89
120 min	1.13	0.02	0.13	0.46	0.20	nd	nd	nd	nd	nd	1.94	0.00	1.94
SEM	1.05	0.04	0.06	0.40	0.23	0.03	-	0.45	0.02	0.01	1.78	0.54	2.28
*P*-value													
Linear	<0.001	<0.001	<0.001	<0.001	<0.001	<0.001	-	<0.001	<0.001	<0.001	<0.001	<0.001	<0.001
Quadratic	0.001	0.19	0.006	0.04	0.003	0.73	-	<0.001	0.01	0.55	0.002	<0.001	<0.001

^a^ *PRO* progoitrin, *EPRO* epi-progoitrin, *GNL* gluconapoleiferin, *GNA* gluconapin, *GBN* glucobrassicanapin, *SNB* sinalbin, *GST* gluconasturtin, *4-OHGBS* 4-hydroxyglucobrassicin, *GBS* glucobrassicin, *NGBS* neoglucobrassicin, *Alk* alkenyl glucosinolates, *Ara + Ind* aralkyl plus indolyl glucosinolates, *nd* not detected, *SEM* standard error of the means. Alkenyl glucosinolates: PRO, EPRO, GNL, GNA, GBN; aralkyl glucosinolates: SNB, GST; indolyl glucosinolates: 4-OHGBS, GBS, NGBS

### Amino acids content

The amino acid content is reported in Table 3. There was a linear decrease (*P* < 0.05) in the content of alanine, aspartic acid, glutamic acid and glycine with increasing toasting time. Increasing toasting time also caused a linear decrease (*P* < 0.001) of the lysine and arginine content. Arginine and lysine contents were reduced by 7 and 23 %, respectively, after toasting for 120 min in comparison with the untoasted RSM. The content of OMIU-RL was also reduced (*P* < 0.001) linearly with increasing toasting time. After 120 min of toasting, the OMIU-RL content was 38 % lower than that in the untoasted sample. The reduction of the OMIU-RL content after toasting was more pronounced than the reduction of the lysine content. This is reflected in the reduction of the OMIU-RL to lysine ratio from 0.98 in the untoasted RSM sample to 0.80 in the RSM toasted for 120 min.

**Table 3:** Amino acid contents (g/16 g N) and ratio between OMIU-RL and lysine in rapeseed meal samples toasted for different times^a^

Toasting time	Indispensable amino acids										Dispensable amino acids					
	Arg	His	Ile	Leu	Lys	OMIU-RL	Phe	Thr	Val	Ratio	Ala	Asp	Glu	Gly	Ser	Tyr
0 min	5.47	2.93	4.11	7.04	6.31	6.20	4.07	4.67	5.38	0.98	4.63	7.38	17.34	5.33	4.48	3.30
20 min	5.50	2.95	4.17	7.13	6.08	5.66	4.12	4.70	5.43	0.93	4.68	7.39	17.45	5.38	4.56	3.34
40 min	5.42	2.93	4.13	7.06	5.83	5.32	4.06	4.67	5.38	0.91	4.64	7.35	17.30	5.33	4.51	3.26
60 min	5.18	2.85	4.03	6.90	5.46	4.85	3.97	4.55	5.28	0.89	4.53	7.15	16.83	5.19	4.39	3.17
80 min	5.23	2.91	4.08	6.97	5.39	4.61	4.03	4.59	5.34	0.86	4.58	7.21	17.05	5.25	4.45	3.24
100 min	5.25	3.01	4.18	7.15	5.23	4.22	4.12	4.68	5.47	0.81	4.69	7.36	17.40	5.38	4.55	3.27
120 min	5.08	2.94	4.09	6.99	4.85	3.86	4.02	4.61	5.38	0.80	4.60	7.18	17.06	5.27	4.47	3.23
SEM	0.04	0.01	0.02	0.03	0.13	0.21	0.02	0.02	0.02	0.02	0.02	0.03	0.07	0.02	0.02	0.02
*P*-value																
Linear	<0.001	0.09	0.09	0.15	<0.001	<0.001	0.37	0.15	0.07	<0.001	0.05	0.02	0.03	0.04	0.06	0.35
Quadratic	0.72	0.24	0.23	0.24	0.74	0.32	0.29	0.17	0.17	0.92	0.14	0.30	0.16	0.16	0.12	0.16

^a^ *Arg* arginine, *His* histidine, *Ile* isoleucine, *Leu* leucine, *Lys* lysine, *OMIU-RL* O-methylisourea reactive lysine, *Phe* phenylalanine, *Thr* threonine, *Val* valine, *Ratio* ratio OMIU-RL to lysine, *Ala* alanine, *Asp* aspartic acid, *Glu* glutamic acid, *Gly* glycine, *Ser* serine, *Tyr* tyrosine, *SEM* standard error of the mean

### Secondary protein structure

The proportion of intermolecular β-sheets tended (*P* = 0.06) to be affected by the quadratic effect of toasting time (Table 4). This proportion markedly increased after the initial 20 min of toasting and thereafter gradually decreased with longer toasting times. In contrast, the proportion of α-helix decreased by half after the initial 20 min of toasting and increased thereafter with toasting time from 16.2 % after 20 min to 19.7 % after 120 min toasting. Toasting time had a quadratic effect (*P* = 0.04) on the α-helix proportion. Linear (*P* = 0.04) and quadratic (*P* = 0.01) effects of toasting time were noticed on the T2 proportion. This element increased after the first 20 min toasting, but stabilize thereafter. Linear (*P* < 0.001) and quadratic (*P* = 0.004) effects of toasting time were also found for the proportion of A2 elements (Table 4). The increase in these elements was more apparent after the first 20 min of toasting than thereafter.

**Table 4:** Proportion (%) of the secondary structures of rapeseed meals toasted for different times^a^

Toasting time	Intermolecular β-sheets	Intramolecular β-sheets	α-helix	T2	A2	α-helix : β-sheet
	(1,627 – 1,630/cm)^b^	(1,634 – 1,635/cm)	(1,655 – 1,656/cm)	(1,674/cm)	(1,692/cm)	
0 min	49.0	5.5	30.5	8.9	6.1	0.62
20 min	63.1	nd	16.2	13.7	6.9	0.26
40 min	60.8	nd	17.5	14.4	7.2	0.29
60 min	60.0	nd	18.0	14.6	7.4	0.30
80 min	59.4	nd	18.0	15.0	7.5	0.30
100 min	57.5	nd	20.4	14.6	7.5	0.36
120 min	57.9	nd	19.7	14.7	7.7	0.34
SEM	1.0	-	1.1	0.5	0.1	0.03
*P*-value						
Linear	0.94	-	0.63	0.04	<0.001	0.56
Quadratic	0.06	-	0.04	0.01	0.004	0.03

^a^ *T2* turns, *A2* intermolecular hydrogen-bonded β-sheets, *nd* not detected, *SEM* standard error of the mean

^b^Regions of the Fourier transform infrared spectra

### Degradation rate constants

Indolyl, alkenyl and total glucosinolates had the highest degradation rate constants and the shortest half-life compared to the other parameters (Table 5). The degradation rate constant of indolyl glucosinolates was 2-fold higher than that of alkenyl glucosinolates. The half-life of total glucosinolates was approximately 5.5-fold lower than that of OMIU-RL and 10-fold lower than that of lysine. The degradation rate constant of OMIU-RL was almost twice that of lysine. The NSI had a degradation rate constant 2-fold as low as that of PDI, which is also reflected in a longer half-life.

**Table 5:** Degradation rate constants and half-life (first order reactions) of parameters measured after toasting of rapeseed meal^a^

Parameter	Degradation rate constant, ×10^−3^/min	Half-life, min
Enthalpy of denaturation	6.1	114
NSI	4.8	144
PDI	10.6	65
Alkenyl glucosinolates	20.4	34
Indolyl glucosinolates	44.3	16
Total glucosinolates	22.3	31
Arginine	0.7	990
Lysine	2.1	330
OMIU-reactive lysine	3.8	182

^a^ *NSI* nitrogen solubility index, *PDI* protein dispersibility index

### *In vitro* CP digestibility

With the two-step enzymatic digestibility method, there was a tendency (*P* = 0.08) for a linear increase of the *in vitro* dry matter digestibility along with toasting time (Table 6). In addition, there was a quadratic effect of toasting time on the *in vitro* CP digestibility (*P* = 0.005), increasing before 60 min of toasting and decreasing thereafter. With the pH-STAT enzymatic hydrolysis method, there were no effects of toasting time on the DH after 120 min hydrolysis. However, the rate of protein hydrolysis was linearly (*P* < 0.001) reduced with increasing toasting time.

**Table 6:** Coefficients of *in vitro* digestibility and hydrolysis of rapeseed meal samples toasted for different times^a^

Toasting time	Two-step enzymatic digestibility		pH-STAT enzymatic hydrolysis	
	CDMD	CCPD	DH 120 min	*k* (/s)
0 min	0.382	0.752	0.186	0.029
20 min	0.393	0.758	0.173	0.032
40 min	0.406	0.773	0.179	0.027
60 min	0.418	0.776	0.175	0.024
80 min	0.415	0.764	0.183	0.018
100 min	0.411	0.753	0.179	0.017
120 min	0.415	0.750	0.185	0.013
SEM	0.005	0.003	0.002	0.002
*P*-value				
Linear	0.08	0.32	0.23	<0.001
Quadratic	0.19	0.005	0.18	0.47

^a^ *CDMD* coefficient of dry matter digestibility, *CCPD* coefficient of crude protein digestibility, *DH* degree of hydrolysis, *SEM* standard error of the mean

The *in vitro* CP digestibility with the two-step enzymatic method did not correlate with any of the parameters of protein changes measured. In contrast, significant correlations were found between *k* and NSI (*r* = 0.88, *P* < 0.001), PDI (*r* = 0.79, *P* = 0.001), lysine (*r* = 0.92, *P* < 0.001), OMIU-RL content (*r* = 0.91, *P* < 0.001), and the proportion of A2 in the secondary structure (*r* = −0.74, *P* = 0.004). Significant correlations were also found between the DH after 120 min hydrolysis and the proportion of intermolecular β-sheets (*r* = −0.66, *P* = 0.01), α-helices (*r* = 0.60, *P* = 0.03) and the ratio of α-helices to β-sheets (*r* = 0.58, *P* = 0.04) in the secondary structure.

## Discussion

The small reduction in the enthalpy of denaturation and the NSI, along with a high ratio of OMIU-RL to total lysine (0.98) in the untoasted RSM can be considered indicators of a RSM with low protein denaturation and high protein nutritional quality. A decrease in protein solubility in heat-treated materials is an indication of the aggregation of proteins after denaturation [24, 25]. As more proteins become denatured and unfolded with increasing toasting time, intra and intermolecular interactions within and between proteins promote aggregation.

Both NSI and PDI have been used before as indicators of the extent of thermal damage in processed protein-rich ingredients (e.g. soybean meal and RSM) [26–28]. Protein solubility and the standardized ileal digestibility of AA in cecectomized broilers were reduced with increasing autoclaving time of a commercial RSM [28]. Pastuszewska et al. [29] suggested that rapeseed meals with a NSI in 0.5 % KOH between 55 and 60 % can be considered of a high nutritional value. These values were achieved in our experiment between 40 and 60 min toasting, which correspond to toasting times used during commercial RSM production [29].

The increasing NDF and ADIN contents with increasing toasting time in the present experiment was previously described after hydrothermal treatments of canola and RSM [8, 29, 30]. These authors, however, also reported an increase in the ADF content, which was not found in the present study. The difference in the results could be due to milder conditions used in the present experiment compared to those reported previously. The increase of the ADIN content was linked to a decrease of the standardized ileal protein digestibility and was proposed as a good indicator for protein damage [30]. Although it has been suggested that heat treatment increases the linkage between proteins and fiber [29], it is possible that the increase in the content of NDF, ADF and ADIN results from the inability of the solvents used to solubilize the aggregated and chemically modified proteins (e.g. melanoidins) [30].

The changes observed in protein denaturation and solubility with increasing toasting time do not parallel the changes observed in the secondary structure of proteins. Contrary to what we expected, there was an increase in the proportion of α-helix and a decrease of intermolecular β-sheets with increasing toasting time after the initial 20 min of toasting. Previous research [7, 20] described a decrease in the proportion of α-helix and an increase in that of intermolecular β-sheet structures after thermal treatment, which was also expected in the present experiment with increasing toasting time. The increase in the proportion of intermolecular β-sheets was linked to a decrease in the *in vitro* CP digestibility [7]. It is possible that with increasing denaturation, which is the rate limiting step, there is partial unfolding of the proteins with a simultaneous increase of aggregation and (partial) refolding of the secondary structure. Most of the literature on thermal-induced changes to the secondary structure of proteins reports the effects after a certain period of time (e.g. autoclaving at 120 °C for 20 min) [7, 20, 31], but do not include the changes occurring during that time period. When considering all time points analyzed in the present study, the net results for secondary structure are still comparable to the results described in literature after autoclaving [7, 20, 31]. The presence of A2 bands has been related to aggregation of proteins due to intermolecular hydrogen-bonded anti-parallel β-sheets [32] or to absorption of infrared light from the amino acid side chains [7].

The formation of Maillard reaction products (MRP) results from chemical changes to AA, for which the most susceptible ones are lysine and arginine [33]. With increasing toasting time, chemical changes of AA continue to occur resulting in the formation of more early MRP and the conversion of the early into advanced MRP and melanoidins [33]. In contrast to early MRP, advanced MRP cannot be reverted into lysine under conditions of 6 mol/L acid hydrolysis [15]. This was noticed by a decrease of lysine content with increasing toasting time. The decrease of the OMIU-RL to lysine ratio is probably the result of higher rate of formation of early MRP compared to advanced ones. Previous research only showed a reduction in the ratio between lysine and reactive lysine after 64 min of toasting [8]. This might be due to the low reactive lysine to lysine ratio determined already in the rapeseed cake (i.e. 0.81). In a recent experiment [3], values of lysine for commercial RSM of German oil mills ranged from 5.5 to 5.3 g/100 g CP, which correspond in our experiment to toasting times of approximately 71 and 91 min, respectively. However, in that same experiment, the OMIU-RL content ranged from 4.4 to 4.0 g/16 g N for the same RSM. This makes the ratio of OMIU-RL to lysine much lower compared to those reported here. The variation in the results could be due to the shorter incubation times for the reaction with OMIU used in the those studies [3, 8] compared to the longer incubation times used in the present study (2–2.5 vs. 7 d). It is possible that proteins with a large extent of thermal damage and high aggregation (low solubility) might need longer incubation times for the OMIU reactive to penetrate within the aggregate structure and bind with the free lysine. Alternatively, free lysine may have been formed during toasting, which cannot be analyzed by the OMIU-RL procedure.

The decrease in the ratio lysine to CP with thermal treatments has been reported before [8, 30, 34–36]. According to these authors, lower ratios, as compared to higher ones, indicate that protein damage occurred due to the formation of MRP. A decrease of this ratio led to a decrease of the ileal digestibility of CP and AA [8, 30, 34]. Therefore, the decrease in the lysine to CP ratio reported in our experiment with increasing toasting time is indicative of protein damage and could lead to a decrease of the *in vivo* protein digestibility. The ratio lysine to CP of the 0 min toasted RSM in the present study (6.3 g lysine/100 g CP) corresponds well to values previously reported for non-toasted canola meal [36]. The apparent ileal digestibility of lysine of the non-toasted canola meal in broilers ranged from 87 to 92 % [36]. A lower ratio of lysine to CP (5.55 g lysine/100 g CP) was reported in that study for solvent-extracted canola meals from 7 different production plants. The apparent ileal digestibility of lysine for the solvent-extracted canola meals was lower and more variable (ranging from 65.5 to 85.7 %) than the values reported for non-toasted canola meal [36]. Other authors [8, 30] have reported lysine to CP ratios of 5.2 g/100 g CP in commercial canola meals and 5.1 g/100 g CP in RSM toasted for 48 min, indicating damage of the proteins. These values corresponded to standardized ileal digestibilities of lysine in growing pigs of 68.2 and 64 %, respectively. In the present experiment, values of lysine to CP ratio that resemble the ones reported by these authors were obtained after 100 min toasting, indicating that the thermal treatments applied by these authors were likely more severe than the ones used herein.

First order reactions have been used previously to model the decrease in glucosinolate content of red cabbage [37] and reactive (available) lysine in model systems [38]. One of the aims of toasting during the production of RSM is to inactivate the glucosinolates without affecting the nutritional quality of proteins (e.g. lysine content). Glucosinolates were degraded at a faster rate than the degradation of OMIU-RL and lysine. Furthermore, the rate constant of decrease of the solubility parameters (i.e. NSI and PDI) is higher than that of OMIU-RL and lysine. This could be an indication that changes in the structure of proteins occur earlier during toasting than chemical (i.e. Maillard) changes. The higher rate constant of decrease of PDI could make it a better indicator of the changes in solubility after toasting of RSM than NSI. Previous research in soybeans indicated that PDI reflects protein quality better than NSI, especially after processing at mild conditions [39].

The range of values obtained with the two-step *in vitro* CP digestibility can be considered as narrow (75.0–77.6 %). A linear decrease of the *in vitro* CP digestibility from 71 % in the 48 min toasted RSM to 62 % in the 93 min toasted meal was reported in a recent study [8]. This also matched the reported decrease in standardized ileal CP digestibility in that study. Toasting time did not affect the DH after 120 min indicating that the observed protein changes are not a restriction for protein hydrolysis when the enzymes are available sufficiently long to hydrolyze. However, the rate at which the enzymes access the substrate during hydrolysis was linearly reduced by toasting time. A reduction in the rate of hydrolysis with increasing heating time has been reported before [40] for glycinin from soybeans. The high correlation of the rate of hydrolysis with protein solubility (in alkali and water), and lysine or OMIU-RL contents could explain the decrease in the rate of hydrolysis with increasing toasting time. It is not possible, however, to distinguish if the formation of aggregates (i.e. lower solubility) or the chemical modification of the Maillard-sensitive AA is the major factor controlling the rate of hydrolysis, as both occur simultaneously during toasting. The decrease in the rate of protein hydrolysis with increasing toasting time could explain the reduction of the ileal protein digestibility reported in other studies after toasting [5, 8].

Extensive reviews have suggested inclusion levels of total glucosinolates ranging from 2 to 2.5 μmol/g diet for pigs, whilst for poultry, the inclusion level ranges from 2 to 10 μmol/g diet [41, 42]. To maintain these total glucosinolates level, at the maximum rate of protein hydrolysis (i.e. 20 min of toasting) in this study, the inclusion level of RSM in the diets for pigs and poultry can be 9.8 and 49 %, respectively. This would also involve a loss of 4 % lysine and 9 % OMIU-RL with respect to the untoasted RSM. At the maximum *in vitro* CP digestibility (i.e. 60 min of toasting) in this study, the inclusion level in the diets for pigs can increase to 18.2 %, whilst there would be no limit to the inclusion level for poultry diets. This would involve a loss of 13 % lysine and 22 % OMIU-RL with respect to the untoasted RSM. However, the inclusion level of rapeseed or canola meal in the diets for pigs might depend not only on the content of total glucosinolates, but also on the type of glucosinolates included [43]. Whilst the feed intake of weanling pigs did not decrease after the inclusion of 2.2 μmol/g diet of total glucosinolates from Brassica napus [44], the inclusion of 2.2 μmol/g diet of total glucosinolates from Brassica juncea in diets for growing-finishing pigs resulted in a decrease of feed intake and weight gain [43]. The major glucosinolate in B. juncea is gluconapin, whilst B. napus contains higher levels of progoitrin than gluconapin [45].

## Conclusions

Toasting of RSM for increasing time induces physical and chemical changes to the proteins and affects its nutritional value. These changes are correlated to the rate of protein hydrolysis but not the *in vitro* CP digestibility or the extent of hydrolysis. Degradation of glucosinolates occurs earlier during toasting and at higher rates than that of OMIU-RL and lysine.

## Abbreviations

AA: Amino acids
CP: Crude protein
DH: Degree of hydrolysis
DM: Dry matter
MRP: Maillard reaction products
NSI: Nitrogen solubility index
OMIU: *O*-methylisourea
PDI: Protein dispersibility index
RSM: Rapeseed meal
